# New concepts in neurology education: successful implementation of flipped classroom lectures

**DOI:** 10.1186/s42466-022-00196-7

**Published:** 2022-08-08

**Authors:** Katharina Mosene, Henrik Heitmann, Dennis Pötter, Friederike Schmidt-Graf

**Affiliations:** 1 TUM Medical Education Center, School of Medicine, Technical University of Munich (TUM), Munich, Germany; 2 Department of Neurology, Klinikum Rechts der Isar, Technical University of Munich (TUM), Ismaninger Straße 22, 81675 Munich, Germany; 3 Department of Psychosomatic Medicine and Psychotherapy, Technical University of Munich (TUM), Munich, Germany

**Keywords:** e-learning, Flipped classroom, Teaching, Neurology education, Neurology lectures, COVID-19

## Abstract

In order to inspire and attract young people to Neurology, we must offer high-quality and attractive teaching! To improve neurological education at our Medical School (Technical University of Munich), we converted the main lecture into an e-learning concept using a flipped classroom model. Students had to prepare with a video and a text as well as answering multiple choice questions before each lecture. As a further incentive, students with ≥ 80% right answers in multiple choice questions received a bonus for the final exam. During the lectures, predominantely patient cases were discussed to apply, improve and enhance the previously acquired knowledge. The realignment of the main lecture in Neurology into a flipped classroom model was very successful and was further optimized in the following semesters based on the evaluations obtained for the new concept. Moreover, this enabled us to quickly switch to remote teaching during the COVID-19 pandemic, while still offering lectures of high quality. In addition, this new teaching concept attracts students for Neurology. Furthermore, the exemplary conversion of the Neurology main lecture to a flipped classroom concept also serves as best practice and motivation to adapt other courses in our faculty and far beyond.

## Letter to the editor

Even before the COVID-19 pandemic, there was increasing emphasis on improving teaching and training of medical students [1, 2]. This is crucial, especially in a complex subject like Neurology [3]. On the one hand to enhance the understanding of neurological symptoms and clinical syndromes, which is of increasing importance in clinical care and science, and on the other hand to attract personnel for our field in view of the increasing need for medical staff [4, 5].

**Fig. 1:**
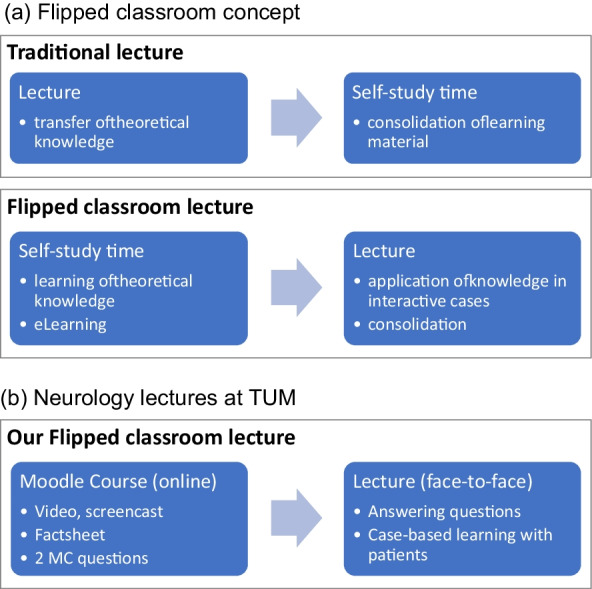
**a** Flipped classroom concept. **b** Neurology lectures at TUM

In 2018, our realignment of the main lecture in Neurology at our Medical School at the Technical University of Munich (TUM) to an e-learning concept with flipped classroom aimed at improving the quality of teaching (possibility of individual, self-organized preparation, more active in-person lectures, focus on knowledge transfer and training of competencies) (Fig. 1) [1, 2, 6, 7]. Greater flexibility through asynchronous learning, independent of location and time, support of innovative learning concepts and active participation by students are just some factors underlining the attractiveness of e-learning [5, 8]. In addition, the new method should attract students to Neurology.

A video or screencast was created for each of the 23 Neurology lectures (23 topics, Table 1), as well as a suitable text (textbook chapter, review, etc.) and corresponding multiple choice (MC)-questions for knowledge testing were provided in an online Moodle-course prior to the face-to-face event as preparation. The time for preparation was intended to be around 45 min and the factual knowledge was required for attendance of the following lectures. Each of the lectures began with answering open questions, after which the focus was set on a case-based processing of knowledge using patient cases. In this small and large group work, discussions and problem-solving-oriented as well as case-based thinking were the focus. Using online or live surveys, knowledge could be checked in real time or opinions could be assessed.

**Table 1:** Lecture topics

Introduction, Metabolic diseases of the CNS
Autoimmune diseases of the CNS 1: Multiple sclerosis
Autoimmune diseases of the CNS 2: limbic encephalitis, paraneoplastic diseases
Infections of the CNS: meningitis, encephalitis (e.g. lyme disease, HIV, zoster)
Dizziness, vertigo
Primary headache
Neuropathic pain, facial pain
Differential diagnoses spinal syndromes
Cerebrovascular diseases 1: ischemic stroke
Cerebrovascular diseases 2: hemorrhagic stroke
Cranial nerve syndromes
Hypokinetic movement disorders 1: IPS
Hypokinetic movement disorders 2: APS
Hyperkinetic movement disorders: tremor, dystonia
Sleep disorders, RLS, narcolepsy
Diseases of the peripheral nervous system and muscles
Dysimmune neuropathies
Motor neuron disease
Neuromuscular diseases: myasthenia, lambert eaton syndrome
Chorea, ataxia
Neurooncology
Epilepsy
Irreversible loss of brain function

Detailed evaluations were obtained from students and lecturers. As regularly also in former times, students anonymously provided a grade and comments for every lecture. Additionally, we conducted a questionnaire-based survey for students and for every lecturer.

According to the evaluations obtained for the first flipped classroom semester, 85.5% used the preparatory material regularly before the lecture. Moreover, 87.7% found the materials very helpful or helpful. This led to a deeper understanding of the topic as reported by 83.3%. Due to the information provided in advance and the case-based approach, 85.7% found the topics more comprehensible. Additionally, 87.2% recommended attending the lectures and 78% preferred the new form of teaching. Specifically, students named the overall high quality of the content, the interactive concept and a better understanding as the most positive aspects in their comments. In contrast, they criticized the partially too extensive preparation materials including low text quality as well as too much repetition of factual knowledge in lectures in some cases. The overall evaluation yielded a grade between 1 and 2 (average 1.79 on a scale from 1 = “very good” to 6 = “unsatisfactory”) for all lectures including the preparation materials. The lecturers indicated that 93.3% of students were prepared well to acceptable. Active participation in the discussion rated very good to good by 87.6%. Additionally, students achieved better grades in the final exam (average 2.16) compared to previous semesters, and for the first time no student failed.

After these experiences in the first semester, the necessary improvements in the selection of the text material for preparation and the didactic concept of the face-to-face lectures were implemented for the following semester. Especially the preparation materials had to be optimized, considering the duration of the preparation. To this end, we established a "fact sheet" with a maximum of 2 pages instead of any text. An incentive system was created to motivate students: answering correctly more than 80% of all MC-questions throughout the semester led to a bonus for the final exam. In addition, an online exam coaching was established, in which the approach to MC-questions and important factual knowledge are repeated. These optimizations were not only reflected by the very positive evaluations. Moreover, students achieved an even better average grade of 1.58 the final exam in this semester.

In 2020, the COVID-19 pandemic not only forced us to an immediate digitization of teaching but also significantly advanced it [8, 9]. In this context, we were in an excellent position thanks to the previously described realignment: only small adaptations had to be made such as converting the face-to-face events to zoom-meetings. This enabled us to offer high-quality teaching even without face-to-face events, which was reflected by excellent evaluations and enthusiasm for Neurology by the students. During the pandemic the percentage of students actively participating in the course further increased. In 2020, an average of 91% used the fact sheet, 79% saw the preparation video and 35–81% (average 62.5%) accessed the recorded lecture, with 46–112/200 students participating in the zoom-meetings. Moreover, 96% took part in the MC-survey and 82.2% received the bonus points.

E-learning has become an integral part of our everyday life. However, high-quality teaching with e-learning formats requires time-consuming planning to develop a dedicated and sophisticated concept as it should not just be a 1:1 online implementation of previous materials [1, 10]. Well thought-out new concepts should be implemented rather than “emergency remote teaching”. Our concept for flipped classroom lectures is very successful and continues to serve as a role model in the faculty and beyond. Optimizing education holds the potential to stimulate the interest in our field and will thus help us to attract sufficient numbers of personnel for Neurology in the future [5]!

## Data Availability

The dataset is available from the corresponding author on reasonable request.

## Abbreviations

COVID-19: Coronavirus disease 2019
MC: Multiple choice
